# Supplementary material for: Overlapping cell population expression profiling and regulatory inference in C. elegans
Source: BMC Genomics. 2016 Feb 29;17:159. doi: 10.1186/s12864-016-2482-z (PMC4772325; doi:10.1186/s12864-016-2482-z)
Supplement: Additional file 13: — Web supplement. (DOC 21 kb) [file 12864_2016_2482_MOESM13_ESM.zip › sortWeb/clusters/hier.300.clusters/28.html]

Cluster 28 

## Cluster 28

### Expression

| cnd-1 rep. 1 | cnd-1 rep. 2 | cnd-1 rep. 3 | pha-4 rep. 1 | pha-4 rep. 2 | pha-4 rep. 3 | ceh-27 | ceh-36 | ceh-6 | F21D5.9 | mir-57 | mls-2 | pal-1 | pros-1 | ttx-3 | unc-130 | hlh-16 | irx-1 | ceh-6 (+) hlh-16 (+) | ceh-6 (+) hlh-16 (-) | ceh-6 (-) hlh-16 (+) | cnd-1 singlets | pha-4 singlets | 0 | 60 | 120 | 150 | 180 | 240 | 330 | 390 | 420 | 480 | 540 | 570 | 600 | 630 | 660 | NAME | Functional description |
| --- | --- | --- | --- | --- | --- | --- | --- | --- | --- | --- | --- | --- | --- | --- | --- | --- | --- | --- | --- | --- | --- | --- | --- | --- | --- | --- | --- | --- | --- | --- | --- | --- | --- | --- | --- | --- | --- | --- | --- |
|  |  |  |  |  |  |  |  |  |  |  |  |  |  |  |  |  |  |  |  |  |  |  |  |  |  |  |  |  |  |  |  |  |  |  |  |  |  | ZK1248.17 |  |
|  |  |  |  |  |  |  |  |  |  |  |  |  |  |  |  |  |  |  |  |  |  |  |  |  |  |  |  |  |  |  |  |  |  |  |  |  |  | F57B1.13 |  |
|  |  |  |  |  |  |  |  |  |  |  |  |  |  |  |  |  |  |  |  |  |  |  |  |  |  |  |  |  |  |  |  |  |  |  |  |  |  | F44A2.11 |  |
|  |  |  |  |  |  |  |  |  |  |  |  |  |  |  |  |  |  |  |  |  |  |  |  |  |  |  |  |  |  |  |  |  |  |  |  |  |  | F29F11.19 |  |
|  |  |  |  |  |  |  |  |  |  |  |  |  |  |  |  |  |  |  |  |  |  |  |  |  |  |  |  |  |  |  |  |  |  |  |  |  |  | F49H6.16 |  |
|  |  |  |  |  |  |  |  |  |  |  |  |  |  |  |  |  |  |  |  |  |  |  |  |  |  |  |  |  |  |  |  |  |  |  |  |  |  | F48B9.12 |  |
|  |  |  |  |  |  |  |  |  |  |  |  |  |  |  |  |  |  |  |  |  |  |  |  |  |  |  |  |  |  |  |  |  |  |  |  |  |  | K10D6.7 |  |
|  |  |  |  |  |  |  |  |  |  |  |  |  |  |  |  |  |  |  |  |  |  |  |  |  |  |  |  |  |  |  |  |  |  |  |  |  |  | D1081.15 |  |
|  |  |  |  |  |  |  |  |  |  |  |  |  |  |  |  |  |  |  |  |  |  |  |  |  |  |  |  |  |  |  |  |  |  |  |  |  |  | C34D1.14 |  |
|  |  |  |  |  |  |  |  |  |  |  |  |  |  |  |  |  |  |  |  |  |  |  |  |  |  |  |  |  |  |  |  |  |  |  |  |  |  | Y39A3A.4 |  |
|  |  |  |  |  |  |  |  |  |  |  |  |  |  |  |  |  |  |  |  |  |  |  |  |  |  |  |  |  |  |  |  |  |  |  |  |  |  | ZK1225.4 |  |
|  |  |  |  |  |  |  |  |  |  |  |  |  |  |  |  |  |  |  |  |  |  |  |  |  |  |  |  |  |  |  |  |  |  |  |  |  |  | ZC15.11 |  |
|  |  |  |  |  |  |  |  |  |  |  |  |  |  |  |  |  |  |  |  |  |  |  |  |  |  |  |  |  |  |  |  |  |  |  |  |  |  | W03G11.13 |  |
|  |  |  |  |  |  |  |  |  |  |  |  |  |  |  |  |  |  |  |  |  |  |  |  |  |  |  |  |  |  |  |  |  |  |  |  |  |  | *srab-5* | Serpentine Receptor, class AB (class A-like) |
|  |  |  |  |  |  |  |  |  |  |  |  |  |  |  |  |  |  |  |  |  |  |  |  |  |  |  |  |  |  |  |  |  |  |  |  |  |  | T07F12.9 |  |
|  |  |  |  |  |  |  |  |  |  |  |  |  |  |  |  |  |  |  |  |  |  |  |  |  |  |  |  |  |  |  |  |  |  |  |  |  |  | ZC47.11 |  |
|  |  |  |  |  |  |  |  |  |  |  |  |  |  |  |  |  |  |  |  |  |  |  |  |  |  |  |  |  |  |  |  |  |  |  |  |  |  | W07G4.10 |  |
|  |  |  |  |  |  |  |  |  |  |  |  |  |  |  |  |  |  |  |  |  |  |  |  |  |  |  |  |  |  |  |  |  |  |  |  |  |  | B0379.2 |  |
|  |  |  |  |  |  |  |  |  |  |  |  |  |  |  |  |  |  |  |  |  |  |  |  |  |  |  |  |  |  |  |  |  |  |  |  |  |  | T07G12.14 |  |
|  |  |  |  |  |  |  |  |  |  |  |  |  |  |  |  |  |  |  |  |  |  |  |  |  |  |  |  |  |  |  |  |  |  |  |  |  |  | R12B2.11 |  |
|  |  |  |  |  |  |  |  |  |  |  |  |  |  |  |  |  |  |  |  |  |  |  |  |  |  |  |  |  |  |  |  |  |  |  |  |  |  | *srh-32* | Serpentine Receptor, class H |
|  |  |  |  |  |  |  |  |  |  |  |  |  |  |  |  |  |  |  |  |  |  |  |  |  |  |  |  |  |  |  |  |  |  |  |  |  |  | F55G7.1 |  |
|  |  |  |  |  |  |  |  |  |  |  |  |  |  |  |  |  |  |  |  |  |  |  |  |  |  |  |  |  |  |  |  |  |  |  |  |  |  | Y73F8A.15 |  |
|  |  |  |  |  |  |  |  |  |  |  |  |  |  |  |  |  |  |  |  |  |  |  |  |  |  |  |  |  |  |  |  |  |  |  |  |  |  | Y113G7A.2 |  |
|  |  |  |  |  |  |  |  |  |  |  |  |  |  |  |  |  |  |  |  |  |  |  |  |  |  |  |  |  |  |  |  |  |  |  |  |  |  | F49C12.15 |  |
|  |  |  |  |  |  |  |  |  |  |  |  |  |  |  |  |  |  |  |  |  |  |  |  |  |  |  |  |  |  |  |  |  |  |  |  |  |  | Y80D3A.8 |  |
|  |  |  |  |  |  |  |  |  |  |  |  |  |  |  |  |  |  |  |  |  |  |  |  |  |  |  |  |  |  |  |  |  |  |  |  |  |  | F43H9.3 |  |
|  |  |  |  |  |  |  |  |  |  |  |  |  |  |  |  |  |  |  |  |  |  |  |  |  |  |  |  |  |  |  |  |  |  |  |  |  |  | D1054.17 |  |
|  |  |  |  |  |  |  |  |  |  |  |  |  |  |  |  |  |  |  |  |  |  |  |  |  |  |  |  |  |  |  |  |  |  |  |  |  |  | F09E8.1 |  |
|  |  |  |  |  |  |  |  |  |  |  |  |  |  |  |  |  |  |  |  |  |  |  |  |  |  |  |  |  |  |  |  |  |  |  |  |  |  | E03H4.3 |  |
|  |  |  |  |  |  |  |  |  |  |  |  |  |  |  |  |  |  |  |  |  |  |  |  |  |  |  |  |  |  |  |  |  |  |  |  |  |  | T25C12.8 |  |

### Phenotypes enriched

none found

### Anatomy terms enriched

none found

### GO terms enriched

none found

### Expression clusters enriched

none found

### Motifs enriched

|  |  |  |  |  |  |
| --- | --- | --- | --- | --- | --- |
| **Motif** | **Logo** | **Possible orthologs** | **Number of motifs in cluster** | **Enrichment** | **FDR corrected p** |
| NR4A2\_si |  | nhr-6 | 26 | 1.61 | 0.020 |
| GM12878\_ETS1\_HudsonAlpha |  | lin-1 | 5 | 7.34 | 0.037 |
| SRF\_1 |  | unc-120 | 6 | 5.61 | 0.039 |
| Mw154 |  | lin-39 | 7 | 4.51 | 0.045 |

### Correlated (and anti-correlated) transcription factors

|  |  |
| --- | --- |
| **Transcription factor** | **Correlation** |
| ZK673.4 | 0.69 |
| fkh-10 | 0.67 |
| F27D4.6 | 0.62 |
| flh-2 | 0.61 |
| ttx-3 | 0.59 |
| F54F2.9 | 0.58 |
| hmg-1.2 | 0.57 |
| syd-9 | 0.56 |
| spr-1 | 0.56 |
| ast-1 | 0.55 |
| ceh-48 | 0.55 |
| ceh-93 | 0.54 |
| nfya-1 | 0.54 |
| hlh-27 | 0.53 |
| W02D7.6 | 0.53 |
| nhr-272 | 0.53 |
| zim-3 | 0.52 |
| nhr-285 | 0.52 |
| nhr-239 | 0.52 |
| C27A12.2 | 0.52 |
| him-8 | 0.52 |
| nhr-276 | 0.52 |
| pqn-21 | 0.51 |
| C34F6.9 | 0.51 |
| daf-19 | 0.51 |
| ZK177.3 | -0.36 |
| mxl-3 | -0.36 |
| nhr-41 | -0.37 |
| nhr-172 | -0.37 |
| nhr-193 | -0.37 |
| end-1 | -0.37 |
| B0310.2 | -0.38 |
| nhr-33 | -0.39 |
| klf-1 | -0.40 |
| nhr-150 | -0.42 |
| nhr-92 | -0.42 |
| nhr-217 | -0.42 |
| dmd-3 | -0.43 |
| nhr-114 | -0.43 |
| nhr-88 | -0.44 |
| nhr-57 | -0.45 |
| nhr-76 | -0.45 |
| nhr-113 | -0.49 |
| tbx-31 | -0.49 |
| nhr-260 | -0.50 |
| cebp-1 | -0.56 |
| nhr-262 | -0.57 |
| nhr-261 | -0.57 |
| nhr-264 | -0.58 |
| hlh-29 | -0.63 |

### ChIP peaks enriched

|  |  |  |  |  |
| --- | --- | --- | --- | --- |
| **Gene** | **Experiment** | **Number of upstream peaks** | **Enrichment** | **FDR corrected p** |
| elt-3 | ELT-3\_Embryos | 3 | 18.59 | 0.02 |
